# Bone Regeneration after Treatment with Covering Materials Composed of Flax Fibers and Biodegradable Plastics: A Histological Study in Rats

**DOI:** 10.1155/2016/5146285

**Published:** 2016-08-11

**Authors:** Tomasz Gredes, Franziska Kunath, Tomasz Gedrange, Christiane Kunert-Keil

**Affiliations:** Department of Orthodontics, Carl Gustav Carus Campus, TU Dresden, Fetscherstrasse 74, 01307 Dresden, Germany

## Abstract

The aim of this study was to examine the osteogenic potential of new flax covering materials. Bone defects were created on the skull of forty rats. Materials of pure PLA and PCL and their composites with flax fibers, genetically modified producing PHB (PLA-transgen, PCL-transgen) and unmodified (PLA-wt, PCL-wt), were inserted. The skulls were harvested after four weeks and subjected to histological examination. The percentage of bone regeneration by using PLA was less pronounced than after usage of pure PCL in comparison with controls. After treatment with PCL-transgen, a large amount of new formed bone could be found. In contrast, PCL-wt decreased significantly the bone regeneration, compared to the other tested groups. The bone covers made of pure PLA had substantially less influence on bone regeneration and the bone healing proceeded with a lot of connective tissue, whereas PLA-transgen and PLA-wt showed nearly comparable amount of new formed bone. Regarding the histological data, the hypothesis could be proposed that PCL and its composites have contributed to a higher quantity of the regenerated bone, compared to PLA. The histological studies showed comparable bone regeneration processes after treatment with tested covering materials, as well as in the untreated bone lesions.

## 1. Introduction

Autografts are still the gold standard in bone grafting because of immediate availability and high success rate, though their amount and applications are limited due to donor site morbidity and graft resorption [1]. The use of autografts is currently becoming narrower and they are often substituted for likewise efficient bone allografts [2]. Host integration and limited long-term functional capacity still require improvement of bone substitutes [3, 4]. Most allografts can provide vastly superior mechanical stability, which is indispensable for therapy of extensive bone damage, caused by traumatic injury, degenerative disease, or tumor resection [5]. In case of severe bone fractures or bone damage, the use of pins, nails, screws, or plates is a reliable method of producing rigid internal fixation and a functionally stable fracture site to keep bone fragments together [6–8]. The most commercially used bone plates and screws are made of metallic materials which are not particularly compatible with such noninvasive diagnostic imaging procedure like magnetic resonance imaging (MRI) and computed tomography (CT) because of metal-related artifacts [9]. Although metal plate fixation will be mostly use after complicated bone fracture, they do not remain without disadvantages. Beside possible corrosion and fatigue strength, the rigid metal plates can cause by the stress shielding effect considerable bone atrophy in plated segment, especially a decreased cortical density and mineral content [10]. Many polymers or polymer-based composites are contemplated as an alternative for bone fixation due to their biocompatibility, high strength-to-weight ratio, radiolucency, biofunctionality, and nontoxicity of degradation by-products [11–13]. Though unreinforced polymers are more ductile than metals and ceramics, they are often not stiff enough to be used to replace or retain hard tissues. A better mechanical property, due to strength and stiffness requirements for hard tissue substitution, exhibits polymer-based composites [14]. They have already been multifunctionally applied, for instance, as biosensors, coatings, and load-bearing implants [15]. Biodegradable composites gain more and more importance for creation of surgical devices which avoid an additional surgery for their removal. The continuous degradation and a gradual load transfer of these materials could stimulate the healing and remodeling of bone tissue [14, 16]. Among the many synthetic biocompatible and biodegradable polymers, polylactide (PLA) and their copolymers have been approved for human clinical uses [17]. PLA has already been used for craniofacial fracture and ankle fixation [17, 18]. PLA and polylactide-co-glycolide (PLGA) are often used for drug delivery, tissue engineering, and manufacturing of medical implants and surgical sutures [19]. PLA occurs in metabolism of all microorganisms and animals incorporated into the tricarboxylic acid cycle; hence, its degradation and excreted products are assumed to be completely nontoxic [20]. Zygomatic fracture fixation with PLA or metal showed similar results; however, 60% of the patients treated with PLA showed intermittent swelling at the implantation site [17, 18].

A very good biocompatibility with bone cells exhibits also polycaprolactone (PCL), used in several biomedical applications, inter alia in scaffolds for bone and cartilage tissue engineering [21]. Owing to the relatively low melting point of PCL, its mechanical properties can be improved in melting techniques by bonding with other polymers or stiffer materials, in the form of particles or fibers [14, 22, 23]. Another group of polymers, polyhydroxyalkanoates, represented by 3-hydroxybutyric acid (PHB) and its copolymers, gained a fixed place in the biomedical field, due to their biocompatibility, biodegradability, and physical and mechanical properties [24, 25]. PHB scaffolds are highly compatible with osteoblast and can induce ectopic bone formation [26]. Recently, it could be shown that PHB membranes can act as matrix for cell migration, proliferation, differentiation, and vascularization in process of bone healing [27]. It was speculated that PHB patches or PHB in form of composites could be an interesting examination object in the treatment of bony defects.

Polymer-based and fiber-reinforced composite materials have been already investigated in animal studies for biocompatible bone defect fillings, adhesion, and anchoring into bone [12, 28, 29]. These materials were reinforced with natural as well as glass or carbon fibers. Flax fibers exhibit better mechanical properties than other natural fibers, comparable to those of glass fibers [30]. Modification of flax fibers to create therapeutic dressing could be of medical interest. One of the first studies on the transgenic flax fibers overproducing various antioxidative compounds has demonstrated promising therapeutic results for a wound dressing [31]. Other genetic modifications of flax plants allowed the synthesis of PHB in the plant fibers which improved their mechanical features and offered thereby an attractive material for industry and medicine [32–34]. This material did not show any inflammation response after subcutaneous insertion and a good* in vitro* and* in vivo* biocompatibility was shown in previous studies [35–37].

Due to preliminary molecular-biological analyses and earlier studies to bone regeneration after usage of PHB, it was hypothesized that composites from transgenic flax plants producing PHB showed faster bone regeneration in comparison with composites of nontransgenic flax plants. The aim of the current study was to both histologically and histomorphometrically, evaluate the effect of polymer-flax composites on the osteogenesis process, using a model of unperforated bone defects at the skull top of rats.

## 2. Material and Methods

### 2.1. Surgical Procedure and Experimental Design

For the study, flax composites were used which have already been described [35–37]. The osteogenic potential of flax composites was investigated in 42 adult Lewis 1A rats (2 months old, body weight between 250 g and 350 g, and of both sexes). The animals were randomly divided into the following 7 groups:

(i) Group 1, controls (*n* = 6): untreated bone defects.
(ii) Group 2, PLA (*n* = 6): bone defects treated with pure PLA-composites.
(iii) Group 3, PLA-transgen (*n* = 6): bone defects treated with composites of PLA and transgenic PHB-producing flax.
(iv) Group 4, PLA-wt (*n* = 6): bone defects treated with composites of PLA and fibers from wildtype flax.
(v) Group 5, PCL (*n* = 6): bone defects treated with pure PCL composites.
(vi) Group 6, PCL-transgen (*n* = 6): bone defects treated with composites of PCL and transgenic PHB-producing flax.
(vii) Group 7, PCL-wt (*n* = 6): bone defects treated with composites of PLA and fibers from wildtype flax.

The approval for all surgical and experimental procedures was issued by the Animal Welfare Committee on the State Government (LALLF M-V/TSD/7221.3-1.1-094/11). All surgical procedures were performed according to standard protocol. This protocol has been published several times [27, 36, 38–40].

In order to compare the data obtained with the molecular-biological findings [35] the skulls were dissected four weeks after composite insertion and fixed in 4% PBS-buffered formalin, dehydrated in a graded series of alcohol, and separately embedded in methylmethacrylate (Technovit 9100 neu, Kulzer, Germany) as previously described [41–44] or in paraffin after decalcification as previously described [40, 45].

### 2.2. Histology

Serial longitudinal sections of about 5 *μ*m were stained with hematoxylin/eosin (HE) for recognizing various tissue types and Masson's trichrome for differentiation between collagen and bone tissue. With Masson's trichrome histological structures were stained as follows: collagen and nonmineralized bone in blue or green, mineralized bone in orange or red, and cell nuclei in dark brown or black [46].

The slices were observed and photographed under alight microscope (BX61, Olympus, Hamburg, Germany) equipped with a calibrated digital camera (Color View II; Soft Imaging System, Olympus Optical GmbH, Hamburg, Germany). Image analysis was performed on composed pictures showing the complete cavity with a magnification of ×100 as previously described [40, 47] using the software cell^∧^F (analySIS Image Processing Olympus, Münster, Germany). From each skull a minimum of 10 sections were histomorphometrically analysed. With this approach we are able to perform an overall conclusion about the bone regeneration in the cavity.

### 2.3. Statistical Analysis

The statistical analyses of variance between groups were made using Mann-Whitney *U* Rank sum test (SigmaStat 3.5 Software, Systat Software, Inc., 1735, Technology Drive, San Jose, CA 95110, USA). Data were given as means ± SEM. *P* < 0.05 was considered statistically significant.

## 3. Results

Wound healing proceeded in all operated animals without any complications and relatively fast. During sampling of bone treated part of calvaria, no signs of inflammation reactions in that tissue could be macroscopically detected.

**Figure 1:**
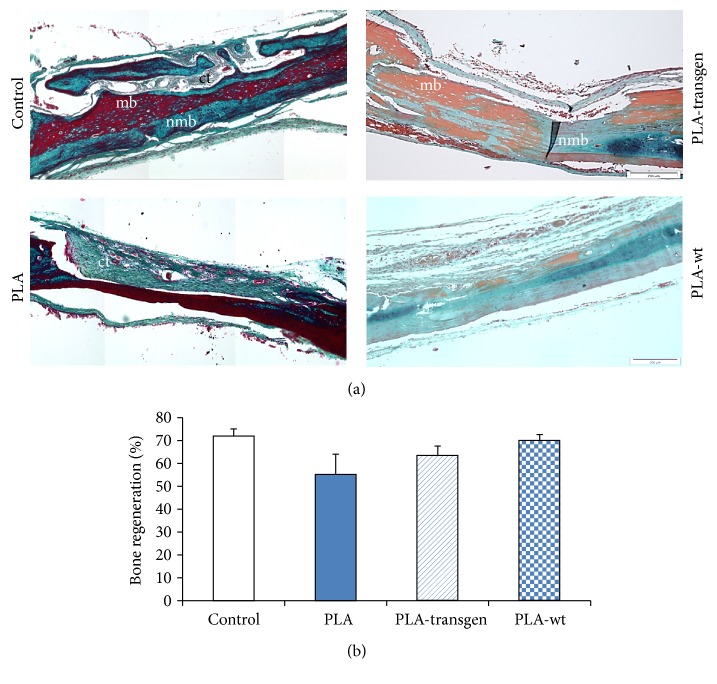
(a) Cranial cross-section embedded in paraffin (control and PLA) or methylmethacrylate (PLA-transgen and PLA-wt) stained with Masson-Goldner four weeks after PLA-composite insertion; collagen and nonmineralized bone in blue or green, mineralized bone in orange or red, and cell nuclei in dark brown or black; (b) histomorphometric analysis of bone regeneration. Stated is mean ± standard error. Bars represent 200 *μ*m. ct = connective tissue; mb = mineralized bone; nmb = nonmineralized bone.

**Figure 2:**
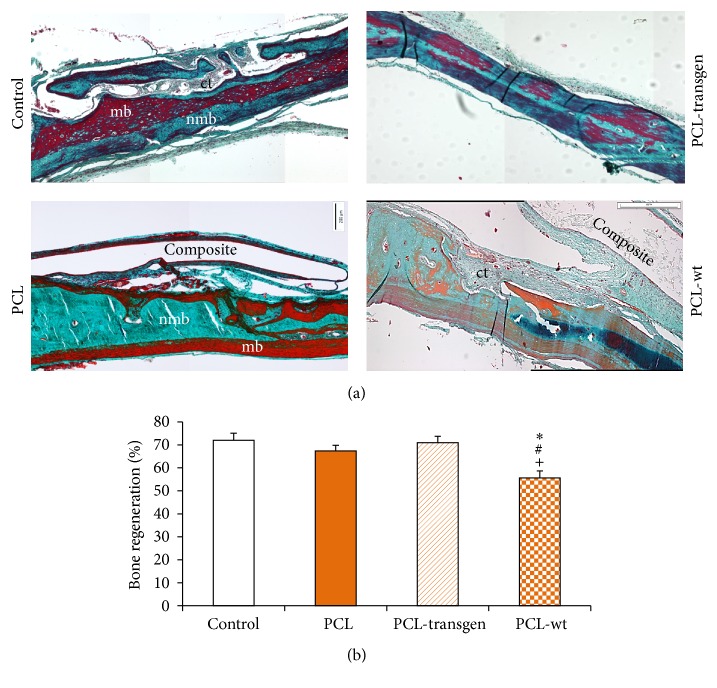
(a) Cranial cross-section embedded in methylmethacrylate (PCL and PCL-wt) or paraffin stained with Masson-Goldner four weeks after PCL-composite insertion; collagen and nonmineralized bone in blue or green, mineralized bone in orange or red, and cell nuclei in dark brown or black; (b) histomorphometric analysis of bone regeneration. Stated is mean ± standard error. Bars represent 200 *μ*m. ^*∗*^ *P* < 0.05 PCL-wt versus control; ^#^ *P* < 0.05 PCL-wt versus PCL; ^+^ *P* < 0.05 PCL-wt versus PCL-transgen. ct = connective tissue; mb = mineralized bone; nmb = nonmineralized bone.

The histological sections showed nearly finished bone healing in untreated bone defects. The surgically created lesions at the beginning of this study were filled after four weeks with nonmineralized bone as well as bone marrow. In addition, a so-called bridging between origin bone and newly formed bone could also be observed (Figures 1(a) and 2(a)).

When using pure PLA, solely connective tissue was detected in the bone defects. In contrast, both flax composites, PLA-transgen and PLA-wt, caused bone regeneration, which was comparable to that of control animals (Figure 1(a)).

In case of bone defects covered with different PCL composites, there was nearly completed bone regeneration with evidence of bone marrow and nonmineralized bone, respectively (Figure 2(a)). Osteolysis and bone resorption did not occur. In addition, it should be noted that all composites are completely embedded in connective tissue in the form of a capsule. When using flax composites, this capsule became thicker (exemplary for PCL and PCL-wt; Figure 2(a)).

Histomorphometric analysis, as shown in Figures 1(b) and 2(b), revealed a regenerated bone mean value of 72.0%  ± 3.1% in untreated control animals. After treatment with PLA composites, the level of bone regeneration was achieved between 55.3 and 70.0%. These results have not shown any statistically significant differences in comparison with bone healing processes in controls. Similar results were obtained after usage of PCL and PCL-transgen, though significantly reduced bone regeneration in bone lesions was found after treatment with PCL-wt (control versus PCL-wt: 72.0 ± 3.1% versus 55.6 ± 3.0, *P* = 0.002, power: 0.931; PCL versus PCL-wt: 67.4 ± 2.4% versus 55.6 ± 3.0, *P* = 0.011, power: 0.737; PCL-transgen versus PCL-wt: 71.0 ± 2.7% versus 55.6 ± 3.0, *P* = 0.003, power: 0.924; Figure 2(b)).

## 4. Discussion 

In the current study, we evaluated the osteogenic potential of new polymer/flax composites in an animal model. Bone histological examination of cavities after treatment with PLA, PCL, or their composites as well as of the empty reference cavities showed spontaneous regeneration of the bony bed, though after four weeks their areas were not completely filled with new bone and they exhibited a large amount of connective tissue, especially after using of pure PLA. The nonsignificant stronger percentage increase of bone regeneration was noticed after using of pure PCL rather than after treatment with PLA. If only the histological preparations were considered, the hypothesis could be proposed that PCL and its composites have contributed to a higher quantity of the regenerated bone, compared to pure PLA.

Biodegradable polymers have already been used for various bone surgical procedures, and in general, they are considered as safe for clinical use [48]. With regard to bone regeneration, many studies have shown that PLA is applicable with satisfactory results as plates, membranes, suture anchors, interference screws, and pins [49, 50]. The adhesive and proliferative behaviors of bone forming cells on various surfaces have been well studied.* In vitro* tests for bone regeneration have demonstrated that rat osteoblasts cultured on PLA retained their phenotype by high expression of alkaline phosphatase activity and collagen synthesis [51]. It was found that additional coating of PLA scaffolds with apatite or apatite/collagen was more efficient for osteoblast-like cells adhesion and proliferation [52]. Further biocompatibility tests of subcutaneously implanted PLA in rat have not detected any acute inflammatory reactions [53, 54]. However, the long-lasting degradation time of PLA can induce late foreign body reactions due to crystalline remnants or a decrease of the pH value during the decomposition [55–57]. Moreover, bone resorption could be observed following degradation of the polymer due to release of nondegraded PLA microparticles [58] or change in the tissue surrounding the degrading PLA implants associated with the leaching of residual monomer or lactic acid [59, 60]. A close contact between the amorphous polymer poly(D,L-lactic acid) and the surrounding tissue without trace of inflammatory tissue, signs of infection, bony necrosis, or any interference of the bone healing process could be recently demonstrated [61]. Our results showed that PLA devices are highly tolerated by host tissues after 4 weeks, which was in agreement with Annunziata et al. [61]. We could affirm a good biocompatibility* in vitro* as well as* in vivo* of PLA and their composites in previous studies [35–37]. The biocompatibility of composites from transgenic flax plants producing PHB did not differ from composites of nontransgenic flax plants and the covering materials composed of flax fibers and PLA or PCL had no influence of the attachment, growth, and survival of the fibroblast cells [37].

PCL degrades much slower than other known biodegradable polymers [62, 63]. It has already been used for bone and cartilage repairs [64] because of its good biocompatibility and high bone inductive potential [65]. A better bone regeneration was achieved using more permeable PCL scaffolds with regular architecture [66]. PCL membranes supported attachment, growth, and osteogenic differentiation of human primary osteoblast-like cells [67]. Previously, it was shown that PCL and PCL-based scaffolds were able to deliver recombinant human bone morphogenetic protein-2 as well as provide sufficient structural support to promote bone healing [68]. A good compatibility of 3D PCL scaffolds was showed, inter alia, in an animal study after insertion of PCL implants into the rat skull with direct contact to the brain. According the investigations of neurogenic potential and neurons, it was demonstrated that PCL did not evoke an undesirable inflammatory response [69]. Moreover, PCL-based scaffolds did not cause further changes to the vascular supply in and around the defect region [70]. In our study, we could also observe a high tolerance of PCL devices by host tissues. Bone healing proceeded without any complications and signs of inflammation. Similar observations were described in other studies [59, 69, 71]. Furthermore, it was found that all composites were completely embedded in connective tissue in the form of a capsule. The encapsulation, as a natural reaction to foreign materials in the body, has also been described previously [59, 72].

In our study, good bone regeneration was observed under the covering materials made of pure polymers and theirs composites. In all cases, a new bone formation was verified by histological examination. In addition, there were no sufficient differences between the controls and treated bone cavities. Recently it was shown that biodegradable PLA membranes, as bone defect coverage, were tested in a sheep model. Enhanced remodeling of the spongiosa into native bony under the membranes could be detected in cranial defects but also without an osteopromoting effect. In contrast to our study, a foreign body reaction around the tested membranes was observed in sheep [73].

Our histological results were partially reflected in the previously published molecular-biological analyses [36]. The significant decrease of expression of 3 genes, which play an important role in bone formation, osteocalcin (Bglap), endopeptidase (Phex), and transcription factor Runx2, might be linked to the reduced amount of new bone formation under cover material of PCL-wt. Furthermore, an unchanged gene expression of Runx2 and Phex was detected after treatment with PLA and its flax composites. Osteocalcin, also called “bone gamma-carboxyglutamate protein,” is a hydroxyapatite-binding protein, which is almost exclusively formed by osteoblasts in large quantities at the beginning of the bone mineralization [74–76]. The differentiation of preosteoblasts is triggered in the progenitor cells involving Runx2 and other transcription factors. It has been shown that a lack of Runx2 can lead to disturbances in the bone formation and mineralization [77]. In knock-out mice with deficiency of Runx2, any ossification processes of the bone tissue were not demonstrated [78]. Phex, a marker for osteocytes, has a significant impact on the transformation of osteoblasts into osteocytes and thus affects the bone mineralization [79, 80].

Based on the presented data, it could be concluded that neither the transgenic nor the native flax fibers can accelerate bone regeneration; however, they did not show any negative influence on new bone formation. This might be attributed, on the one hand, to very low content of flax fibers in the composite (only 20%) and thereby a small proportion of PHB and, on the other hand, to shielding of fibers by embedding in a polymer matrix and to the very slow degradation of used polymers. A further exacerbating factor is that cellulose, main component of flax, is not metabolized and degraded in the body [81, 82]. Therefore, these flax covering materials in presented form are not clinically applicable. Further modifications, for example, the oxidation of cellulose fibers to produce oxycellulose which is absorbable* in vivo* within a short time [83] could have a positive impact on properties of the materials and their osteogenic potential.
